# Cocatalyst loaded Al-SrTiO_3_ cubes for Congo red dye photo-degradation under wide range of light

**DOI:** 10.1038/s41598-023-33249-1

**Published:** 2023-04-18

**Authors:** M. Abd Elkodous, Ahmed M. El-Khawaga, Marwa Mohamed Abouelela, M. I. A. Abdel Maksoud

**Affiliations:** 1 Center for Nanotechnology (CNT), School of Engineering and Applied Sciences, Nile University, Sheikh Zayed, Giza, 16453 Egypt; 2 Chemical Engineering Department, Military Technical College (MTC), Egyptian Armed Forces, Cairo, Egypt; 3 Faculty of Medicine, Galala University, Suez, Egypt; 4 Petrochemical Department, Egyptian Petroleum Research Institute, Cairo, 11727 Egypt; 5 Department of Electrical and Electronic Information Engineering, Toyohashi University of Technology, 1-1 Hibarigaoka, Tempaku-Cho, Toyohashi, Aichi 441-8580 Japan; 6 Radiation Physics Department, National Center for Radiation Research and Technology (NCRRT), Egyptian Atomic Energy Authority (EAEA), Cairo, Egypt

**Keywords:** Environmental sciences, Chemistry, Materials science

## Abstract

The continued pollution, waste, and unequal distribution of the limited amount of fresh water on earth are pushing the world into water scarcity crisis. Consequently, development of revolutionary, cost-effective, and efficient techniques for water purification is essential. Herein, molten flux method was used for the preparation of micro-sized Al-doped SrTiO_3_ photocatalyst loaded with RhCr_2_O_3_ and CoOOH cocatalysts via simple impregnation method for the photo-assisted degradation of Congo red dye under UV and visible irradiation compared with P25 standard photocatalyst. In addition, photoelectrochemical analysis was conducted to reveal the separation and transfer efficiency of the photogenerated e^−^/h^+^ pairs playing the key role in photocatalysis. SEM and TEM analyses revealed that both P25 and the pristine SrTiO_3_ have spherical shapes, while Al-doped SrTiO_3_ and the sample loaded with cocatalysts have cubic shapes with a relatively higher particle size reaching 145 nm. In addition, the lowest bandgap is due to Al^+3^ ion doping and excessive surface oxygen vacancies, as confirmed by both UV–Vis diffuse-reflectance and XPS analyses. The loading of the cocatalysts resulted in a change in the bandgap from n-type (pristine SrTiO_3_ and Al-SrTiO_3_) into p-type (cocatalyst loaded sample) as exhibited by Mott–Schottky plots. Besides, the cocatalyst-loaded sample exhibited good performance stability after 5 cycles of the photocatalytic removal of Congo red dye. OH^·^ radical was the primary species responsible for CR degradation as confirmed by experiments with radical scavengers. The observed performance of the prepared samples under both UV and visible light could foster the ongoing efforts towards more efficient photocatalysts for water purification.

## Introduction

The increasing growth of technology and industry and the rise in living standards have resulted in continual population growth. As a result, a scarcity of numerous resources, especially water is currently a serious issue. According to the World Health Organization, by 2025, half of the world’s population will live in water-stressed regions^1,2^. One strategy for treating wastewater is photocatalytic degradation technology^3,4^. Congo red (CR) is a synthetic and toxic anionic azo dye^5^. Because of its attraction to cellulose fibers, it has many industrial applications, including the wide use in the textile industry^6^. Besides, it is a pH indicator and is used to diagnose amyloidosis. However, CR has a slew of drawbacks. It metabolizes to benzidine, a known human carcinogen. The CR dye is carcinogenic and causes allergic responses and sleepiness. In addition, it can cause respiratory problems, skin, eyes, and gastrointestinal discomfort^7–9^. Various and effective approaches for detoxifying synthetic dyes have been used to treat wastewater pollutants. Due to the activity of photoinduced holes and the reduction capability of electrons, a series of photocatalytic processes begun by semiconductor-based photocatalysts can convert macromolecular organic contaminants into simpler and less hazardous molecular compounds^10^.

Over the past 5 years, our research group has developed and reported many effective nanomaterials and composites for water treatment by the photodegradation of different kinds of water pollutants (organic, inorganic, and heavy metals^11,12^ and the pathogenic microorganisms causing serious human diseases^13^. The materials were based on a layer-by-layer prepared nanocomposite Co_x_Ni_1−x_Fe_2_O_4_; (x = 0.1–0.5–0.9)/SiO_2_/TiO_2_ employing a magnetic core and TiO_2_ photoactive layer separated by a thin silica layer^14,15^. The photocatalytic performance of the prepared nanocomposite was further improved using many methods such as incorporating carbon materials (as electron reservoir) with the TiO_2_ outer layer (dots, rGO, and SWCNTs)^16–18^. In addition, employing cheap plasmonic metals and metal oxides such as Cu was proven to possess good potential^19,20^. Titanate perovskites (MTiO_3_, M = Sr, Ca, Ba) are still receiving momentum due to their notable photocatalytic activities among diverse semiconductor materials. Strontium titanate (SrTiO_3_) is considered the prototype perovskite and has received much interest in photocatalysis. Due to its broadband gap (⁓ 3.2 eV), it has mostly been investigated under UV light for its photocatalytic capabilities^21–23^. However, doping using various methods and metal ions is a typical approach for controlling the band gap width of semiconductors. Many different components have been reported to boost the photocatalytic activity of SrTiO_3_ such as Al, Co, and Cr.

In this study, we investigate the micro-sized Al-doped SrTiO_3_ photocatalyst loaded with RhCr_2_O_3_ and CoOOH cocatalysts for the photo-assisted removal of CR under UV and Vis. light. A molten flux approach was used to prepare the Al-SrTiO_3_ photocatalyst. After that, RhCr_2_O_3_ and CoOOH cocatalysts were loaded onto the Al-SrTiO_3_ photocatalyst via the impregnation method. Obtained and prepared samples were extensively characterized to examine their phase, elemental composition, morphology, structural composition, and optical properties. In addition, the photocatalytic activities against CR in an aqueous phase under UV and Vis light with respect to P25 standard photocatalyst were investigated. In addition, photoelectrochemical analysis was conducted to analyze the separation and transfer efficiency of the photogenerated e^−^/h^+^ pairs for the investigated photocatalysts.

## Materials and methods

### Materials

RhCl_3_·3H_2_O, Co(NO_3_)_2_·6H_2_O, K_2_CrO_4_, SrCl_2_, Al_2_O_3_, SrTiO_3_, and P25 were purchased from Sigma Aldrich, Japan. All chemicals were used as received without any further processing.

### Methods

#### Preparation of Al-doped SrTiO_3_ photocatalyst

Al-SrTiO_3_ photocatalyst was prepared by a molten flux approach as previously reported^24^.

In brief, SrCl_2_, SrTiO_3_, and Al_2_O_3_ with a molar ratio of (10:1:0.02) were placed in an alumina crucible after grinding in an agate mortar. Then, the alumina crucible was heated in a muffle furnace for 10 h at 1150 °C in air. After natural cooling to room temperature, the formed powder was dissociated from the crucible’s wall by ultrasonication. After that, collected powder was washed several times by distilled water (D.W.) to remove any unreacted SrCl_2_. Finally, purified powder was dried in air overnight at 60 °C.

#### Cocatalyst loading

RhCr_2_O_3_ and CoOOH cocatalysts with Rh = 0.1 wt%, Cr = 0.05 wt%, and Co = 0.14 wt% with respect to Al-SrTiO_3_ (main photocatalyst), were loaded onto the main photocatalyst via the impregnation method described by Chen et al.^25^. Firstly, prepared Al-SrTiO_3_ particles (from the previous step) were dispersed in a minimal amount of D.W by ultrasonication for 15 min. Then, calculated volumes of RhCl_3_·3H_2_O, K_2_CrO_4_, and Co(NO_3_)_2_·6H_2_O were added (in order) to the dispersion. After that, water was slowly evaporated from the mixture using a hot plate. Finally, dried powder was calcined in air at 350 °C for 1 h.

#### Characterization of the prepared samples

Formed phase and crystallinity were analyzed by X-ray diffraction (XRD) analysis conducted on Ultima IV X-ray diffractometer (Rigaku, Japan), applying Cu-Kα radiation (λ = 1.54 Å), and operating at 40 kV–30 mA. While morphology of samples, cocatalyst distribution, and purity were revealed via scanning transmission electron microscopy (STEM) using JEM-2100F (JEOL Ltd., Japan), connected with (JED-2300 T) energy-dispersive X-ray (EDX) spectroscopy unit. In addition, diffuse-reflectance and bandgap were measured using V-670 spectrophotometer (JASCO, Japan) to get insights on the optical properties. Colloidal stability and surface charge at different pH values were investigated using ELS-Z1NT analyzer (Photal otsuka electronics, Japan). PL spectra were recorded by (KIMMON KOHA, Japan) laser spectroscopy with an excitation wavelength of 325 nm) to reveal charge recombination affinity. Valence states and elemental structure were revealed by X-ray photoelectron spectroscopy (XPS) analysis on (ULVAC-Phi, QuanteraSXM, Al Kα, Japan). Raman analysis was performed to show the chemical composition of samples using JASCO, NRS-4500 laser Raman spectrometer.

#### Photocatalytic measurements

Photocatalytic degradation of Congo red (CR) aqueous solutions was performed at room temperature (25 °C ± 2 °C). For removal performance because of adsorption, no light (visible or UV) was employed. Basically, a fixed amount of each photocatalyst was added to a known volume of CR anionic dye with a fixed initial concentration at a calculated pH value. While, for the photocatalysis evaluation, dye-containing sample suspensions were irradiated using different light sources (10 W high pressure mercury lamp with a mean wavelength (λ) of 254 nm inside a cylindrical shaped reactor with dimensions of 27 cm length and 2.5 cm diameter and made up of stainless steel. While the visible light was irradiated from a lamb composed of 52 white LEDs (nominal power = 55 W) with 400–800 nm wavelength emission range surrounded by aluminum reflectors to minimize the irradiation loss. The irradiation is applied from the top and the distance between the light and the reactor was fixed at 10 cm.Then, (2 mL) supernatant was withdrawn using a filter-supported syringe (pore size of its filter = 2.5 μm) at fixed time intervals (10 min). After that, the obtained supernatant was more purified using centrifugation to remove any remaining particles of samples. Eventually, the decline in dye concentration due to photodegradation was calculated by a liquid cuvette spectroscopic UV–Vis. analysis (absorbance at λ_max_), via Eq. (1).

1 $${\text{CR degradation }} = C_{{\text{t}}} /C_{{\text{o}}}$$

where *C*_t_ is the concentration at each time interval and *C*_o_ is the initial concentration after adsorption–desorption equilibrium was reached. D.I.W. was used as a reference for measurements.

#### Photoelectrochemical measurements

The investigated photocatalysts were first dispersed in a mixture containing 10% Nafion and equal volumes of isopropyl alcohol and D.W. using ultrasonication for 30 min. Then, 300 µL of the dispersion was deposited on FTO substrate (2 × 3 cm^2^) and left to dry at 60 °C for 1 h. The transient photocurrent response was determined using chronoamperometry measurement at 1.23 V vs. RHE over many on/off cycles using the deposited photocatalyst over FTO substrate, Ag/AgCl (3 M KCl), and Pt wire as the working electrode, the reference electrode, and the counter electrode, respectively. While 0.2 M Na_2_SO_4_ was utilized as an electrolyte. The electrochemical impedance was evaluated under irradiation at the open circuit potential in the frequency range of 10^3^ kHz to 0.01 Hz. The Mott-Schottky plots of the investigated photocatalysts were determined at 10^3^ Hz under dark conditions.

The potential vs. Ag/AgCl converted to the potential vs. RHE using the Nernst equation^26^

2 $$E_{{{\text{RHE}}}} = E_{{{\text{Ag}}/{\text{AgCl}}}} + \, 0.0{\text{59pH }} + E^{0}_{{{\text{Ag}}/{\text{AgCl}}}}$$

where *E*_RHE_ and *E*_Ag/AgCl_ are the potentials vs. RHE and vs. Ag/AgCl, respectively. *E*^0^_Ag/AgCl_ equals 0.197 V at 25 °C.

## Results and discussion

### Characterization of the investigated samples

#### XRD (phase and crystallinity) analysis

**Figure 1:**
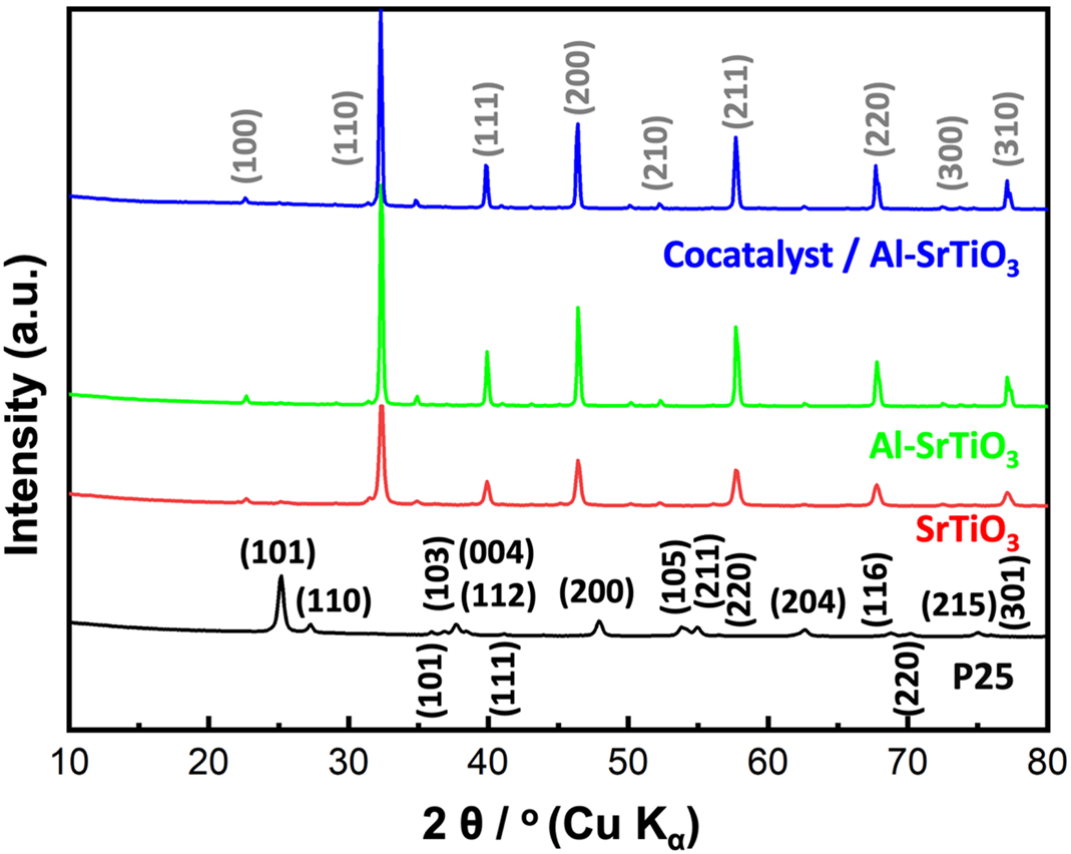
XRD analysis of the investigated samples.

XRD analysis was performed to analyze the phase and crystallinity of the prepared materials^27^ as presented in Fig. 1. For P25 sample, many peaks are recorded at 2θ = 25.3°, 27.5°, 36.2°, 37.2°, 38.4°, 39.1°, 41.5°, 47.5°, 54.6°, 55.8°, 57.1°, 63.8°, 69.2°, 71.58°, 75.6°, and 76.7° corresponding to (101), (110), (101), (103), (004), (112), (111), (200), (105), (211), (220), (204), (116), (220), (215), and (301) crystallographic planes. The detected peaks and their corresponding planes are in a good agreement with those reported for both rutile (JCPDS No. (21-1276)^28–30^) and anatase (JCPDS No. 21-1272^31,32^) phases of TiO_2_ forming the P25^17^. While, for SrTiO_3_ samples, bragg peaks are observed at 2θ = 22.7°, 32.5°, 40.2°, 46.6.5°, 53.3°, 58.6°, 68.2°, 73.7°, and 78.6° corresponding to the (100), (110), (111), (200), (210), (211), (220), (300), and (310) crystallographic planes of the cubic perovskite structure of SrTiO_3_ (JCPDS No. 73-0661)^33^.

It is worth to mention that, neither Al^+3^ ions doping, nor cocatalyst loading could change the crystal structure of SrTiO_3_ (indicated by no change in their XRD patterns), which can be attributed to their lower ratio and/or high dispersion^34^. In addition, all samples possess high crystallinity as shown by their sharp and intense peaks.

#### Raman (chemical composition) analysis

**Figure 2:**
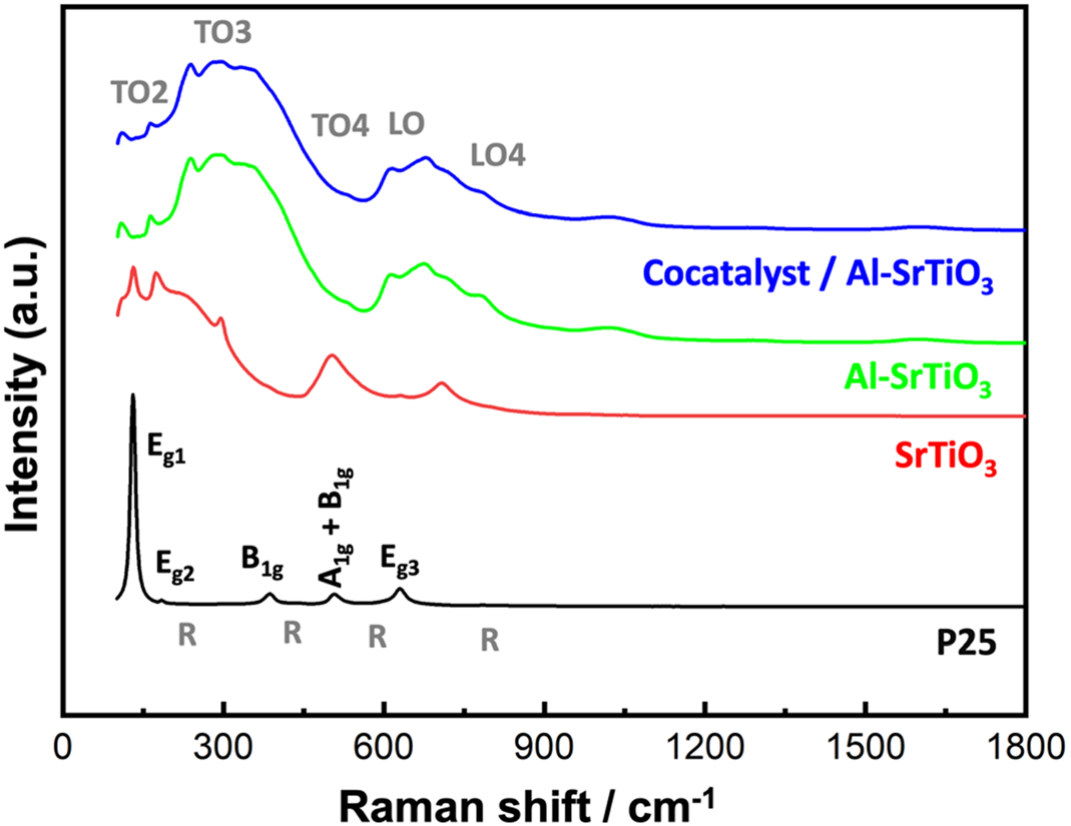
Raman analysis of the investigated samples.

To reveal the chemical composition of the prepared samples, Raman analysis was conducted as depicted in Fig. 2. For P25 sample, sharp and intense Raman peaks corresponding to the Anatase phase of P25 were recorded at 134 cm^−1^ (E_g1_), 175 cm^−1^ (E_g2_), 382 cm^−1^ (B_1g_), 500 cm^−1^ (A_1g_ + B_1g_), and 618 cm^−1^ (E_g3_). The recorded Anatase peaks well matched with those reported in literature^35^. While the corresponding stretching peaks for the rutile phase of P25 are barely detected at 145 cm^−1^ (B_1g_), 443 cm^−1^ (E_g_), 608 cm^−1^ (A_1g_), and 816 cm^−1^ (B_2g_)^36,37^. While for SrTiO_3_ samples, many peaks were detected at 190 cm^−1^, 250–348 cm^−1^, 539 cm^−1^, 621–718 cm^−1^, 786 cm^−1^, which can be assigned to TO2 (O–Sr–O), TO3 (O–Sr–O), TO4 (O–Sr–O), LO (Ti–O–Ti), and LO4 (Ti–O)^38^.

#### UV–Vis. diffuse reflectance and bandgap analyses

UV–Vis. diffuse reflectance spectra of the prepared samples and their corresponding bandgap values are shown in supplementary (S) Fig. 1a and b. For all samples, a strong absorption in the UV range (about 350 nm) is observed, suggesting that UV is a must for their activation. While for SrTiO_3_ samples, blue shift is detected and thus a slight decrease in the calculated bandgap values is noticed, the lowest value is exhibited by the sample loaded with cocatalysts, which is favorable for an improved photocatalytic performance.

#### Colloidal stability and net surface charge: Zeta potential calculations

Zeta potential values of the prepared samples were measured to determine the colloidal stability and to investigate the net surface charge as presented in S. Fig. 2. It is clearly observed that, SrTiO_3_ sample showed the lowest colloidal stability and tendency for agglomeration in all media. In addition, at acidic medium (pH 3), cocatalyst loaded Al-SrTiO_3_ sample is the most stable sample (zeta potential =  + 54.8 mV) with a net positive charge^14^. While at alkaline medium (pH 11), Al-SrTiO_3_ sample possesses the highest stability (−  42.4 mV) with a net negative charge.

#### Photoluminescence (PL) analysis: estimation of charge carrier separation

**Figure 3:**
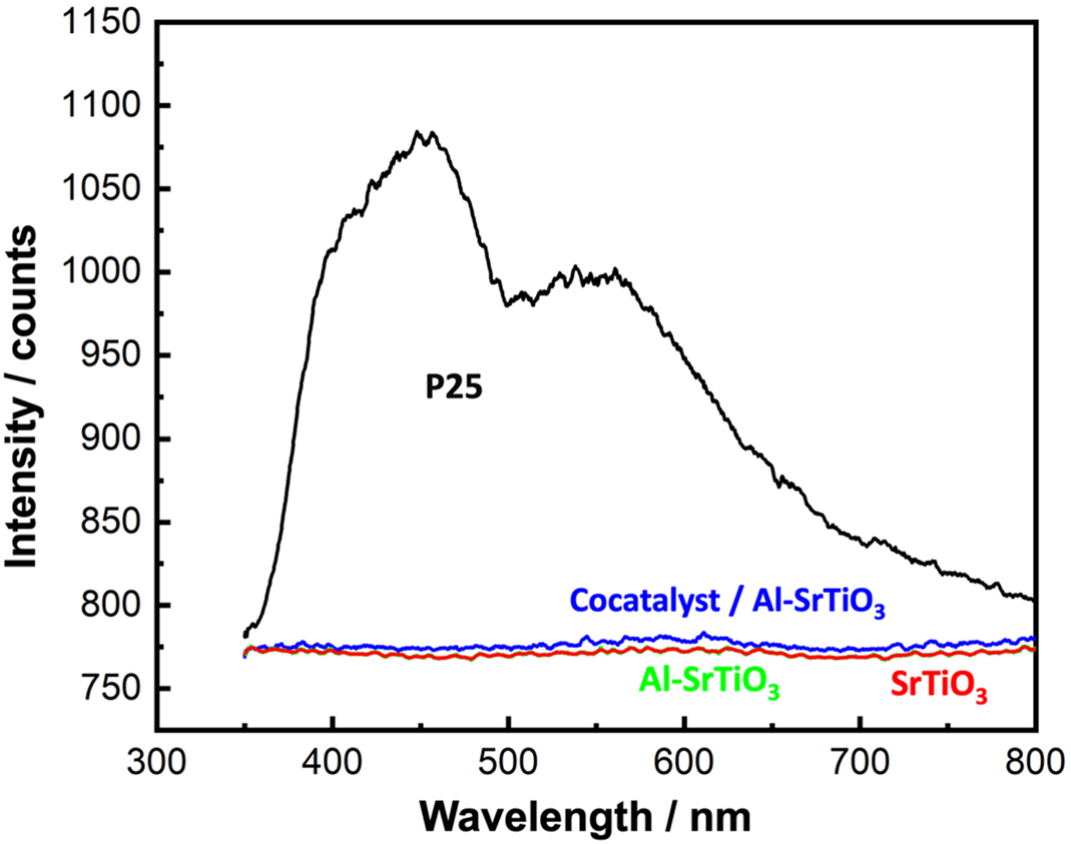
PL analysis of the investigated samples.

To estimate the separation of the photogenerated electron–hole pairs playing a key role in the photocatalytic efficiency, PL analysis was carried out and the results are presented in Fig. 3.

P25 sample showed the highest intensity which means the fastest recombination rate of charge carriers leading to a deterioration of the photocatalytic efficiency^19^. By contrast, SrTiO_3_ samples showed more prolonged separation time of electron–hole pairs confirmed by the significant reduction in their PL intensity, which is a sign of their potential as more efficient photocatalysts. This observation might be due to the doping with Al^+3^ ions which replaced Ti^+3^ acting as a recombination center for charge carriers in Al-SrTiO_3_ sample^39^ or due to the loaded cocatalysts which adjusted the Fermi energy levels in cocatalyst loaded Al-SrTiO_3_ sample^40^ and/or the presence of surface oxygen vacancies acting as electron reservoirs and enhanced the charge separation.

#### XPS (valence states and elemental structure) analysis

**Figure 4:**
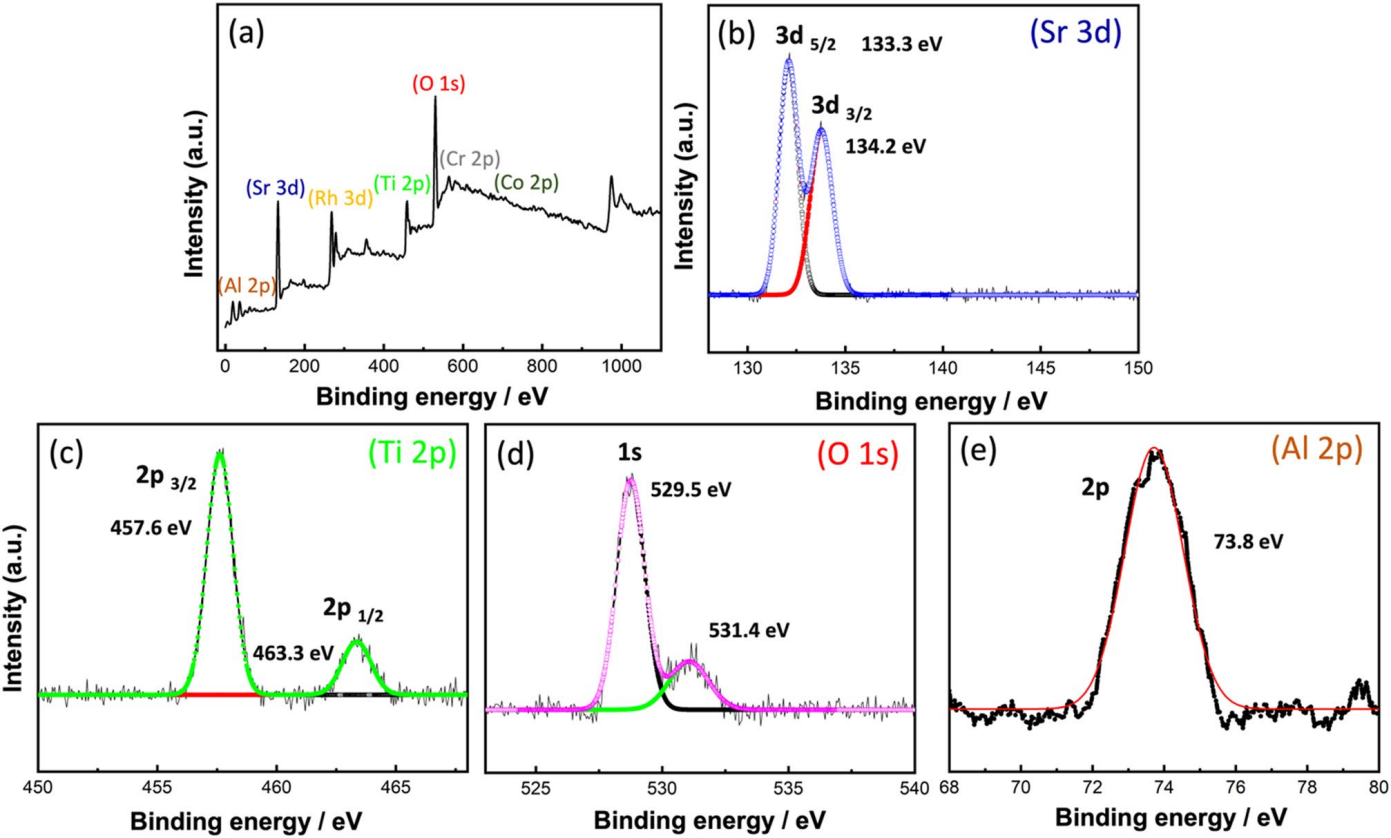
XPS analysis of the prepared cocatalyst loaded Al-SrTiO_3_ sample, (**a**) survey analysis, deconvoluted (**b**) Sr, (**c**) Ti, (**d**) O, and ‘(**e**) Al.

To reveal the valence states and elemental structure of the prepared cocatalyst loaded Al-SrTiO_3_ sample, XPS analysis was carried out as shown in Fig. 4a–d. Figure 4a presents the survey analysis where many peaks corresponding to all constituting elements were observed. While Fig. 4b–e shows the deconvoluted peaks of the main elements (Sr, Ti, O, and Al). For Sr 3*d*, two characteristic peaks at (133.3 eV and 134.2 eV) were detected corresponding to (3*d*_5/2_ and 3*d*_3/2_), respectively indicating the presence of Sr^+2^ state^41^. While for Ti 2*p*, the characteristic two peaks appeared at (457.6 eV and 463.3 eV) corresponding to (2*p*_3/2_ and 2*p*_1/2_), confirm the dominance of Ti^+4^ state^42^. This result also confirms that Ti^+3^ was replaced by Al^+3^ because of the doping process. Ti^+4^ state is more favorable for an improved photocatalytic performance. Regarding O 1*s*, another two peaks were recorded, one at (529.5 eV) of the metal oxides and another at (531.4 eV) indicating the presence of adsorbed water molecules or hydroxyl groups. In addition, the observed shoulder at higher binding energies confirms the presence of surface oxygen vacancies which is consistent with PL observation (Fig. 3)^43^. Finally, for Al 2*p*, a single peak is detected at (73.8 eV) corresponding to the Al^+3^ state^44^. Deconvoluted peaks of Rh and Cr of the loaded cocatalysts are shown in S. Fig. 3.

#### SEM (morphology) analysis

**Figure 5:**
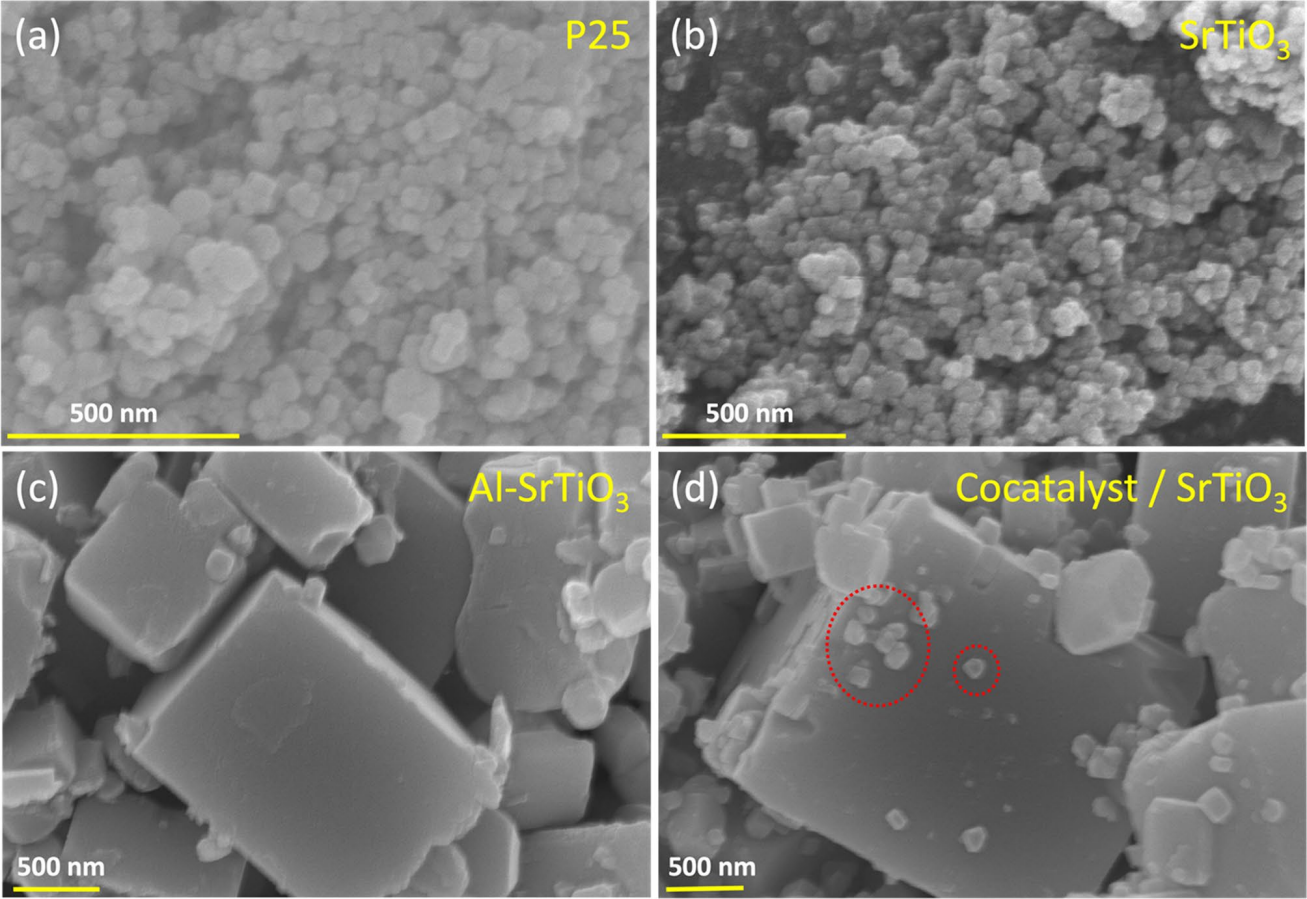
SEM analysis of (**a**) P25, (**b**) SrTiO_3_, (**c**) prepared Al-SrTiO_3_, and (**d**) prepared cocatalyst loaded Al-SrTiO_3_ samples.

According to many recent reports, there is a strong relation between material’s performance and its morphology, that’s why SEM analysis was carried out to reveal the morphology of the prepared samples as shown in Fig. 5a–d. Both P25 and the pristine SrTiO_3_ samples exhibited nearly spherical morphology with relatively smaller diameters compared to both Al-SrTiO_3_ and the cocatalyst loaded Al-SrTiO_3_ samples which possess cubic morphology of the perovskite phase as confirmed by XRD analysis (Fig. 1). For cocatalyst loaded Al-SrTiO_3_ sample, RhCr_2_O_3_ and CoOOH cocatalysts were randomly deposited onto the active sites of Al-SrTiO_3_ (100) and (110) directions of the reductive and oxidative sites because of the impregnation method used for their deposition^25,45^.

#### TEM and STEM mapping analyses

**Figure 6:**
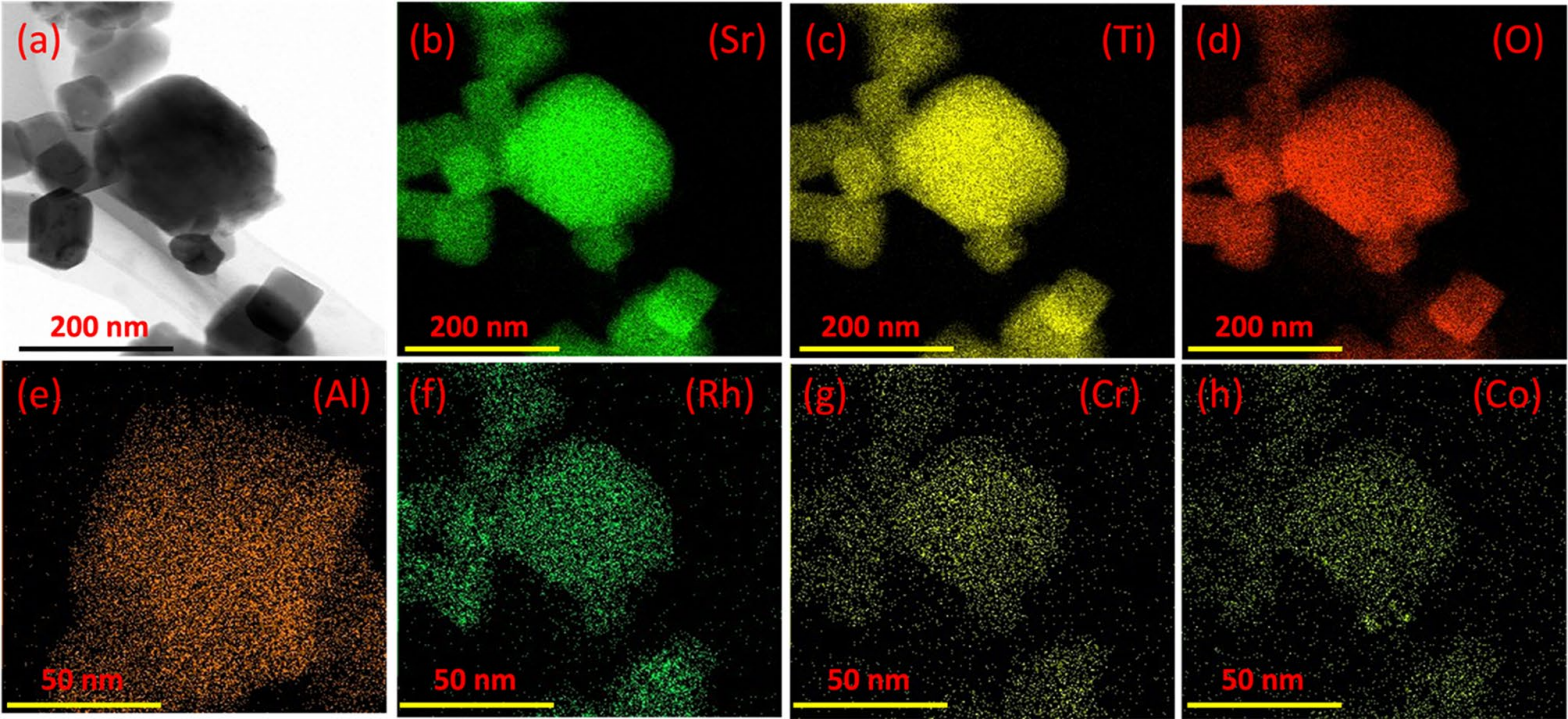
(**a**) TEM analysis and (**b**–**h**) STEM mapping analysis of the prepared cocatalysts over Al-SrTiO_3_ sample.

Morphology, mean diameter, and uniformity distribution of cocatalysts over Al-SrTiO_3_ sample were analyzed using TEM and STEM mapping, respectively as presented in Fig. 6a–h. The calculated mean diameter of particles was about 145 nm which possess cubic shape. While STEM mapping shows the uniform distribution of elements and the cocatalysts over the surface of Al-SrTiO_3_.

### Photocatalytic activity

#### Effect of the employed light source

**Figure 7:**
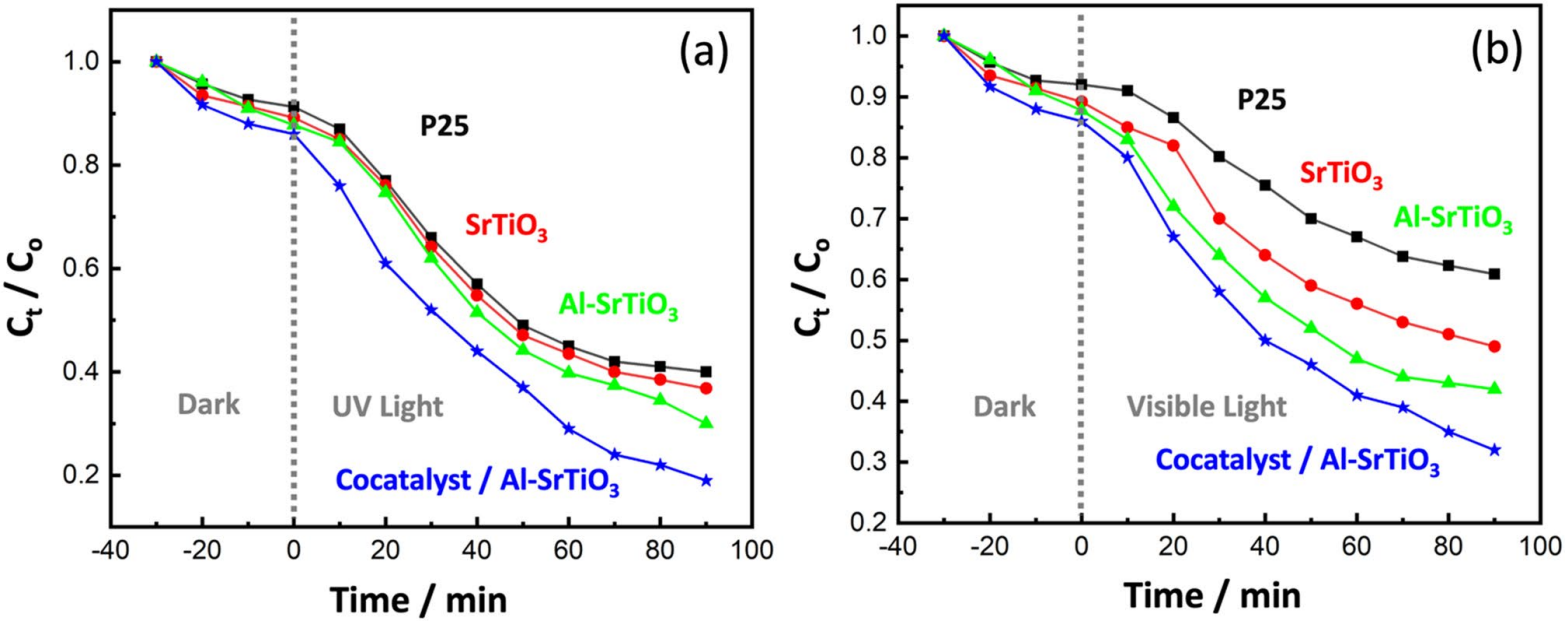
Time-dependent degradation of CR dye in the presence of P25, SrTiO_3_, Al-SrTiO_3_, and cocatalyst loaded Al-SrTiO_3_ catalysts in dark and under (**a**) UV irradiation and (**b**) visible light.

In this section we assessed the photocatalytic activity of P25, SrTiO_3_, Al-SrTiO_3_, and cocatalyst loaded Al-SrTiO_3_ photocatalysts toward CR dye degradation in the dark and under both visible and UV irradiations. The results reveal that the anionic CR dye degrades slightly in the dark, with a removal percentage of just 8.7%, 10.8%, 12.2%, and 14% after 30 min for P25, SrTiO_3_, Al-SrTiO_3_, and cocatalyst loaded Al-SrTiO_3_ samples, respectively. Besides, Fig. 7a shows the time-dependent degradation of CR dye in the presence of P25, SrTiO_3_, Al-SrTiO_3_, and cocatalyst loaded Al-SrTiO_3_ under UV irradiation. The removal percentage of CR dye reached 60% by using the standard P25 photocatalyst, while the removal increased to 63.2%, 70%, and 81% after 90 min by using SrTiO_3_, Al-SrTiO_3_, and cocatalyst loaded Al-SrTiO_3_, respectively. On the other hand, under visible light, the P25, SrTiO_3_, Al-SrTiO_3_, and cocatalyst loaded Al-SrTiO_3_ catalysts exhibited relatively low photocatalytic degradation of CR dye, about 39.1%, 51%, 58%, and 68%, respectively, Fig. 7b. Among all the presented photocatalysts, the cocatalyst loaded Al-SrTiO_3_ has the highest photocatalytic activity for CR dye degradation under both UV and visible light.

#### Effect of catalyst dose and initial dye concentration

Under UV irradiation, the effect of catalytic load on the degradation of CR over the cocatalyst loaded Al-SrTiO_3_ was examined. S. Fig. 4a depicts the degradation efficiency of CR dye (10 ppm) after 90 min of photocatalysis at various catalytic doses ranging from 5 to 20 mg at pH 7. The removal of CR dye using cocatalyst loaded Al-SrTiO_3_ enhanced from 45 to 96% as the catalytic doses increased from 5 to 20 mg. Light scattering and the total active surface area of the cocatalyst loaded Al-SrTiO_3_ sample are both involved in this process^46,47^. In other words, as the number of active sites on the surface of the photocatalyst increases, so will the number of absorbed photons, resulting in more redox reactions and improved efficiency. As a result, additional charge carriers and free radicals are likely to be produced^19^. Gao et al. reported similar behavior in dye degradation by using the cubic SrTiO_3_^48^.

Only a certain amount of adsorbent can adsorb a specific quantity of adsorbate. As a result, the suitable quantity of the adsorbate solution's initial concentration should be quantified. S. Fig. 4b shows the effect of initial CR dye concentration (C_o_ = 5–15 ppm) on the photodegradation performance of the cocatalyst loaded Al-SrTiO_3_ photocatalyst (10 mg at pH 7) under UV light. The percentage of CR dye removed over time decreased with the increase in CR dye's initial concentration. The percentage of CR dye removal by cocatalyst loaded Al-SrTiO_3_ photocatalyst dropped from 90 to 64% when the initial CR dye concentration rose from 5 to 15 ppm. The reduction in the degradation is due to the CR dye constituting an optical filter, whereby it filters photons targeting the photocatalyst and surface saturation with adsorbed CR molecules^5^. The increase in CR dye concentration induces the formation of aggregation on the photocatalyst surface. As a result, just a few photons may penetrate and promote redox reactions, reducing photocatalytic degradation^19^. This result could be explained by the Beer-Lambert Law, which states that an increment in initial dye concentration lowers the path length of photons exiting a solution. This reduces photon absorption by photocatalyst particles, drastically reducing the photocatalytic reaction rate^49^.

#### Reaction kinetics and rate constant

The following equation was utilized to evaluate the first-order kinetics rate for the photocatalytic degradation of CR dye on the cocatalyst loaded Al-SrTiO_3_ photocatalyst:

3 $$\mathrm{ln}\left(\frac{{C}_{t}}{{C}_{^\circ }}\right)=-kt$$

In which k is the reaction rate constant.

S. Fig. 5a depicts the relationship between − ln(C_t_/C_0_) and irradiation time (t) for the degradation of CR dye. The figure demonstrates a significant correlation and correspondence to the pseudo-first-order kinetics, which agrees with the Langmuir–Hinshelwood (L–H) model^50^. All graphs demonstrate a linear relationship between − ln(C_t_/C_0_) and t. The rate constant k for the degradation of CR dye at initial concentrations of 5, 10, and 15 ppm is 0.025, 0.0183, and 0.0109 min^−1^, respectively, via the cocatalyst loaded Al-SrTiO_3_ photocatalyst, S. Fig. 5b.

#### Effect of pH

**Figure 8:**
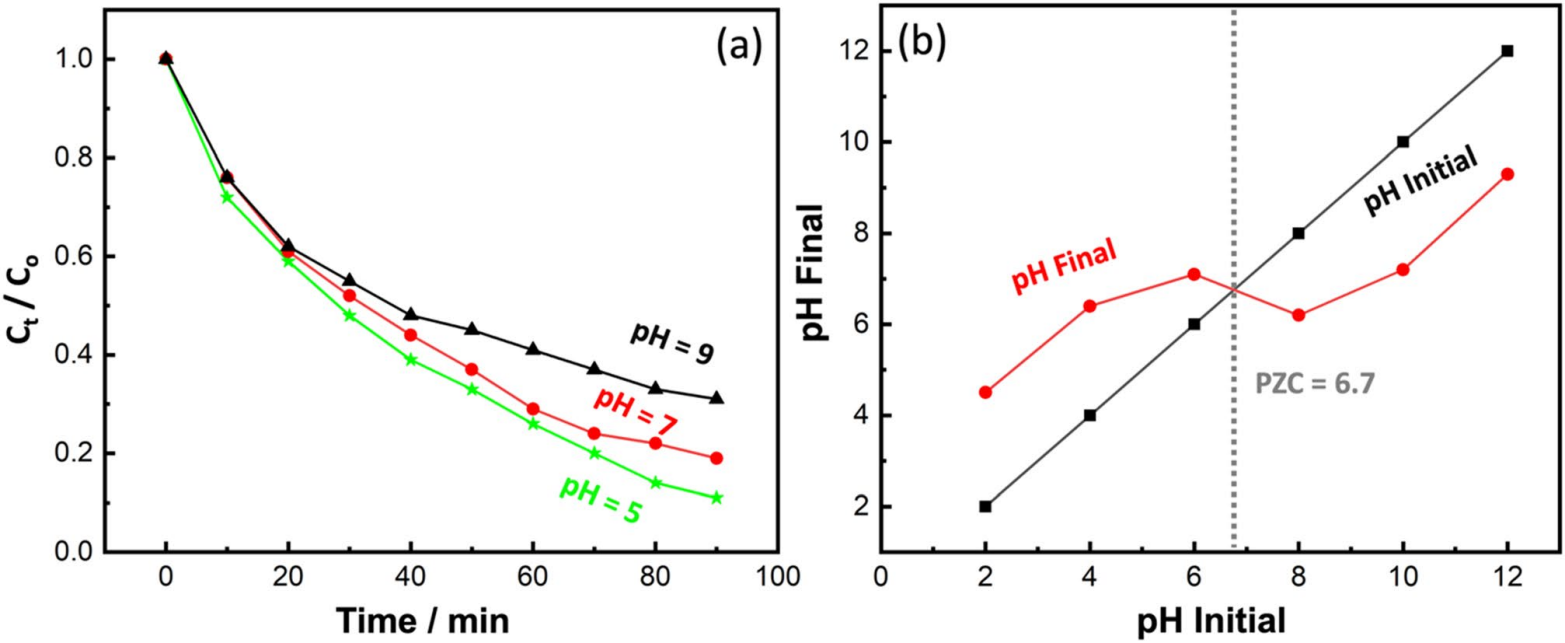
(**a**) Effect of pH value on the degradation percentage of CR by using cocatalyst loaded Al-SrTiO_3_ and (**b**) PZC calculation.

The pH is an essential parameter in CR photocatalytic degradation because it greatly influences the interfacial processes, either anodically or cathodically, by adjusting the charge that is yielded in the catalyst surface^5,51^. The point of zero charge (pH_PZC_) is broadly utilized to explore the influence of pH. The photoactivity of the prepared cocatalyst loaded Al-SrTiO_3_ photocatalyst significantly reduces in an alkaline medium (pH 9), as illustrated in Fig. 8a, owing to the negative surface of cocatalyst loaded Al-SrTiO_3_ in the alkaline medium as confirmed by S. Fig. 2, this causes electrostatic repulsions between the photocatalyst and CR molecules, noticed by the low removal of CR (69% at pH 9). It is worth noting that the CR dye is still stable at this pH. The removal efficiency in the basic medium is ascribed to the repulsion forces between the negative charges of the sulfonate SO_3_^-^ function of CR and the negative surface of cocatalyst loaded Al-SrTiO_3_^52^. At an acidic pH of 5, the photocatalytic effectiveness achieves its maximum with a 89% reduction. This is due to the positive charge of the cocatalyst loaded Al-SrTiO_3_ surface inducing the CR to adsorb on it, enabling an effective photodegradation process. The optimal pH for the CR photooxidation is close to pH_PZC_, which equals 6.7 (see Fig. 8b). On the other hand, at a neutral medium (pH 7), the removal effectiveness achieves its highest value of approximately 81%. This behavior may be due to reducing the cocatalyst loaded Al-SrTiO_3_ surface charge and resulting in enriching CR adsorption. Table 1 shows a comparative study between our results and the published works in the literature.

**Table 1:** Comparative study of the photodegradation of the Congo red dye using cocatalyst loaded Al-SrTiO_3_ and the other photocatalysts.

photocatalyst	Degradation efficiency %	Time, min	Light Source	Ref.
Y_2_O_3_	24	60	UV	^53^
ZnO/Cu_2_O	77	60	Visible light	^54^
NiO	65	30	Sunlight	^55^
PVP/ZnO	97.8	240	UV	^56^
CoFe_2_O_4_	83	90	UV	^57^
CeO_2_–chitosan NPs	86.2%	90	UV	^58^
SnO_2_–Fe_3_O_4_	50.76	90	UV	^59^
Chitosan/BiOCl/ZnO	93	40	UV	^60^
Cocatalyst loaded Al-SrTiO_3_	81	90	UV	This work

#### Effects of H_2_O_2_

Since the function of H_2_O_2_ as a source of OH^**·**^ under UV light, increasing the initial concentration of H_2_O_2_ may accelerate the rate of OH^**·**^ generation. Hence, the removal of CR dye by UV/H_2_O_2_ was thoroughly investigated utilizing various [H_2_O_2_]_0_ concentrations ranging from 10% % to 30% using 10 mg of cocatalyst loaded Al-SrTiO_3_ sample and a constant concentration of CR dye (10 ppm at pH of 7) to determine the best conditions in treating varying levels of CR. The removal of CR dye was considerably enhanced by increasing the concentrations of [H_2_O_2_]_0_. The removal achieved was 83%, 88%, and 95% for 10%, 20%, and 30% of [H_2_O_2_]_0_. Compared to 81% in the absence of [H_2_O_2_]_0_, the increasing concentration of H_2_O_2_ remarkably facilitated the removal of CR dye, as presented in S. Fig. 6. Similar results have also been marked in earlier studies^61–63^.

#### Reuse and recycling

**Figure 9:**
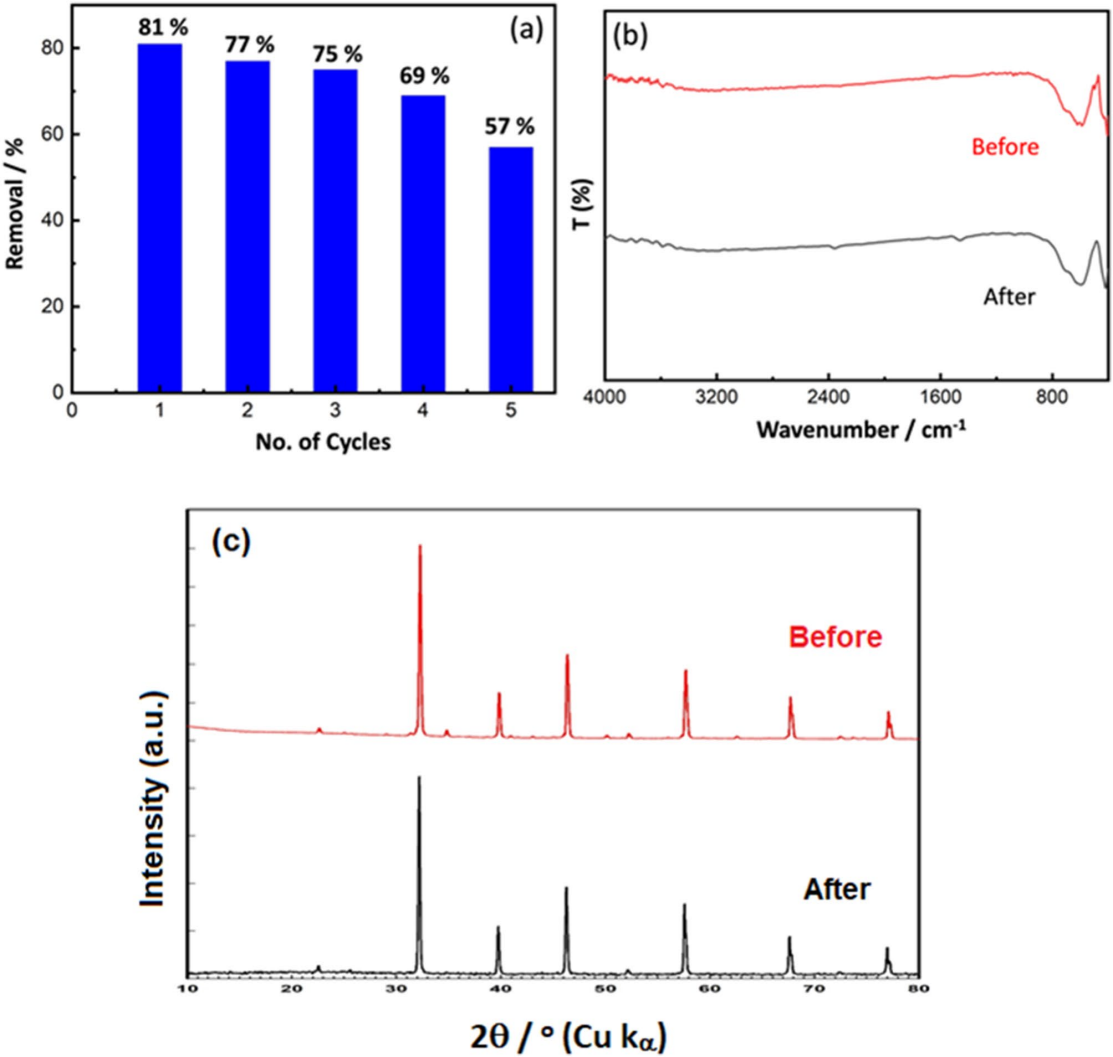
(**a**) Recyclability of cocatalyst loaded Al-SrTiO_3_ for CR degradation under UV irradiation (**b**) FTIR spectra and (**c**) XRD patterns of cocatalyst loaded Al-SrTiO_3_ before and after 5 cycles of CR degradation.

The reuse and recovery of photocatalysts employed in environmental remediation are critical for long-term waste management. As a result, cost-effectiveness, and excellent photocatalytic degradation efficiency of cocatalyst loaded Al-SrTiO_3_ sample were attained. Long-term photocatalytic activity and photocatalyst stability are critical parameters. A photocatalyst's reuse stability is critical to its industrial fields. After one cycle, cocatalyst loaded Al-SrTiO_3_ was collected by centrifugation, washed three times with deionized water, and dried in an electro-thermic blast oven at 100 ◦C for 8 h before being employed in the next cycle. As a result, an additional study was carried out to explore the reuse stability of cocatalyst loaded Al-SrTiO_3_ in the photocatalytic reduction of CR dye under UV irradiation, as illustrated in Fig. 9a. The parameters for the photocatalytic reuse tests were the same as for the photocatalytic activity evaluation described above. The photocatalytic activity of cocatalyst loaded Al-SrTiO_3_ reduced as the cyclic number increased, but such declines are moderate; for example, 69% of CR dye in the solution is still reduced in the fourth cycle. FTIR and XRD were utilized to evaluate the stability of the sample before and after 5 cycles of CR degradation, as shown in Fig. 9b,c. No other impurity band or any foreign XRD peak are existed in the cocatalyst loaded Al-SrTiO_3_ after degradation, and no distinct band disappeared, revealing that the cocatalyst loaded Al-SrTiO_3_ had excellent stability and photocatalytic activity, and no additional reactions resulted in the degradation of CR, which is highly relevant in practical uses.

#### Effect of scavengers on CR degradation

ROS are generated by trapping photogenerated electrons or holes in the solution via several species, especially dissolved oxygen, hydroxyl ions, and water molecules. The essential ROS species include hydroxyl (·OH), superoxide (·O_2_), and singlet oxygen (^1^O_2_). The effect of scavengers on the photocatalytic degradation process was studied to identify the most active species. The isopropanol and benzoquinone were employed to trap OH^·^ and ^−^O_2_^·^, respectively^64–66^.

S. Fig. 7 depicts the efficiency of photodegradation of CR by cocatalyst/Al-SrTiO_3_ in the absence and presence of scavengers at 5 ppm concentration. In the presence of benzoquinone and isopropanol, the efficiency reduced from 81% to roughly 65% and 56%, respectively. When isopropanol was added, the photodegradation of CR dye by the cocatalyst loaded Al-SrTiO_3_ was decreased (56%), suggesting that the OH^•^ radical was the primary species responsible for CR degradation. This result is consistent with that observed in S. Fig. 6. Furthermore, adding benzoquinone lowered the degradation rate by 65%, revealing that the ^−^O_2_· radical played a prominent role in CR degradation^49^.

### Photoelectrochemical analysis

Although the photocatalysis mechanism is different from that of photoelectrocatalysis, which might result in different findings, the photoelectrochemical analysis was conducted to get insights into the behavior of the investigated samples from different perspectives.

**Figure 10:**
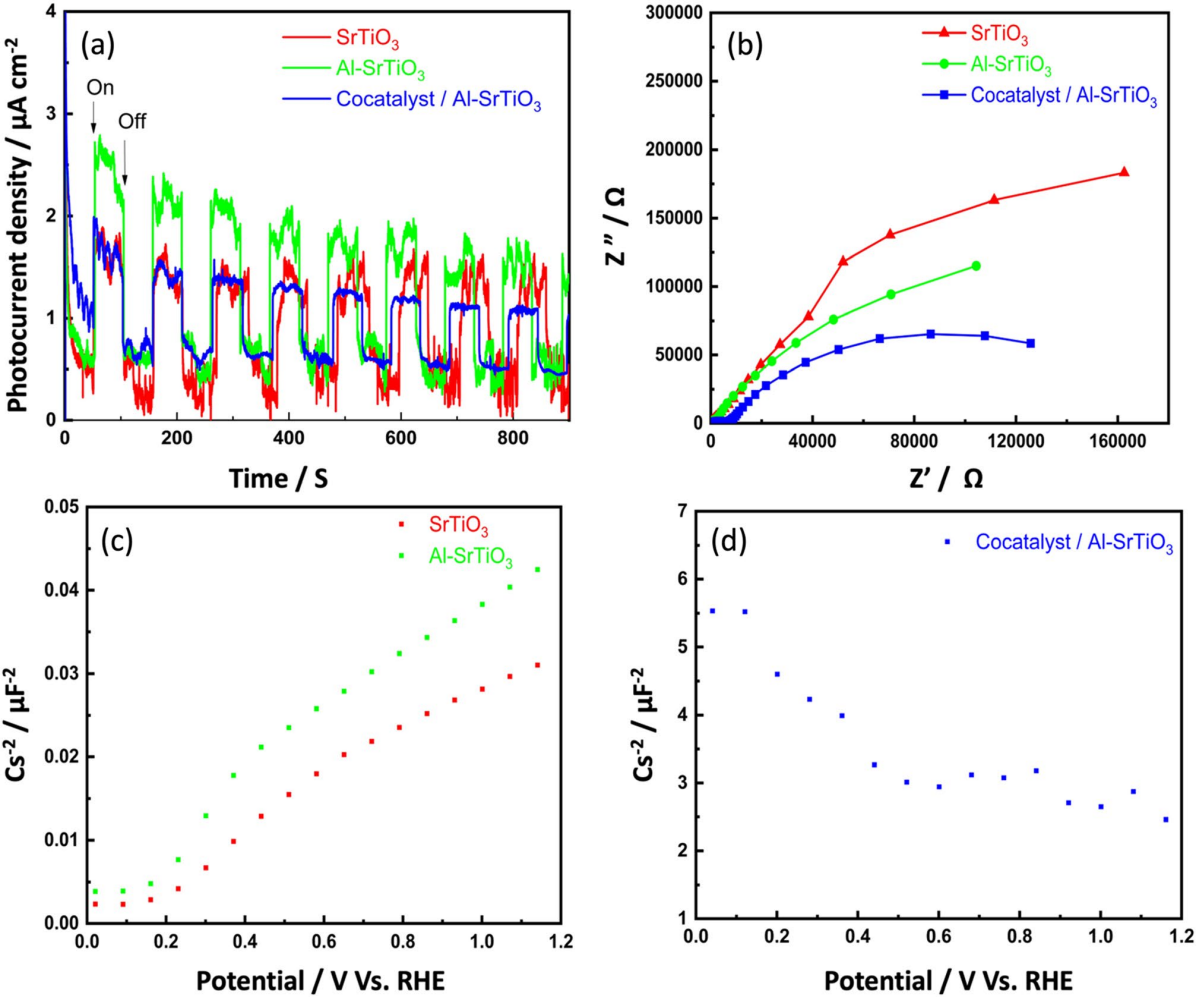
(**a**) Photocurrent response, (**b**) EIS Nyquist curves, and Mott–Schottky plots of (**c**) SrTiO_3_, Al-SrTiO_3_, and (**d**) cocatalyst loaded Al-SrTiO_3_ samples.

The photocurrent response and the EIS measurements were carried out to characterize the separation and transfer efficiency of the photogenerated e^-^/h^+^ for the investigated photocatalysts^67^. Figure 10a shows the transient photocurrent response of SrTiO_3_, Al-SrTiO_3_, and the cocatalyst loaded Al-SrTiO_3_ photocatalysts. It can be observed that under light irradiation, there is a sharp increase in photocurrent, which markedly diminished under darkness. While in Fig. 10b, the arc radius of the semicircle represents the strength of the charge transfer resistance of each photocatalyst. The heterojunction constructed between Al-SrTiO_3_ and the loaded cocatalysts displayed the lowest charge transfer resistance compared to bare SrTiO_3_ and Al-SrTiO_3_ photocatalysts. This result affirmed that the photogenerated e^−^/h^+^ separation efficiency was significantly enhanced due to the loaded cocatalysts, which was favorable for an improved photocatalytic efficiency. In addition, the Mott-Schottky plot clarifies the type of semiconductor materials and their corresponding flat band potential. As displayed in Fig. 10c, SrTiO_3_ and Al-SrTiO_3_ photocatalysts have positive slope corresponding to the n-type semiconductors^68^. It was observed that the flat band potential of SrTiO_3_ was negatively shifted after the incorporation of Al ions, enhancing its photocatalytic activity. While cocatalyst loaded Al-SrTiO_3_ shows a negative slope belonging to p-type semiconductors, Fig. 10d.

On the basis of the preceding results, we propose that CR photodegradation over the cocatalyst loaded Al-SrTiO_3_ occurs via the separation and transfer of photoinduced electron–hole pairs at the heterojunction interface of Al-SrTiO_3_ and the loaded cocatalysts as shown in S. Fig. 8. Wide bandgap of SrTiO_3_ was adjusted and reduced by Al^+3^ doping and cocatalyst loading, upon light activation (E > band gap), electrons are excited from the valence band (VB) to the conduction band (CB), then the loaded cocatalysts promote charge separation, on the reductive active side of Al-SrTiO_3_ (plane 100), the RhCr_2_O_3_ cocatayst reduces CR into final products while at the oxidative active side (plane 110), CoOOH assisted oxidation occurs.

## Conclusion

Molten flux method was used to tailor the bandgap of SrTiO_3_ particles through doping Al^+3^ ions (with one Al ion percent compared with Ti) to prepare an efficient micro-sized Al-SrTiO_3_ photocatalyst by replacing Ti^+3^ recombination centers for charge carriers. To further enhance charge separation/transfer and to lower the activation energy of the prepared photocatalyst, RhCr_2_O_3_ and CoOOH cocatalysts were deposited onto the surface using a simple impregnation method. The photogenerated e^−^/h^+^ separation efficiency was significantly enhanced as confirmed by the EIS measurements. In addition, the separation of the photogenerated charge carriers responsible for improved photocatalytic performance was prolonged, as shown by PL analysis. Furthermore, the n-type Al-SrTiO_3_ became p-type, as shown by the Mott–Schottky plots after cocatalyst deposition. All mentioned factors, along with surface oxygen vacancies as revealed by our XPS investigation, led to the development of an effective photocatalyst that showed promising photo-removal abilities against CR dye with respect to P25 under both UV and visible light. In addition, both structural and performance stabilities after 5 successive cycles of photodegradation were sustained.

## Supplementary Information

Supplementary Information.

## Data Availability

All data generated or analysed during this study are included in this published article [and its supplementary information files].
