# Supplementary material for: Cocatalyst loaded Al-SrTiO3 cubes for Congo red dye photo-degradation under wide range of light
Source: Sci Rep. 2023 Apr 18;13:6331. doi: 10.1038/s41598-023-33249-1 (PMC10113377; doi:10.1038/s41598-023-33249-1)
Supplement: Supplementary file 1 — Supplementary Information. [file 41598_2023_33249_MOESM1_ESM.docx]

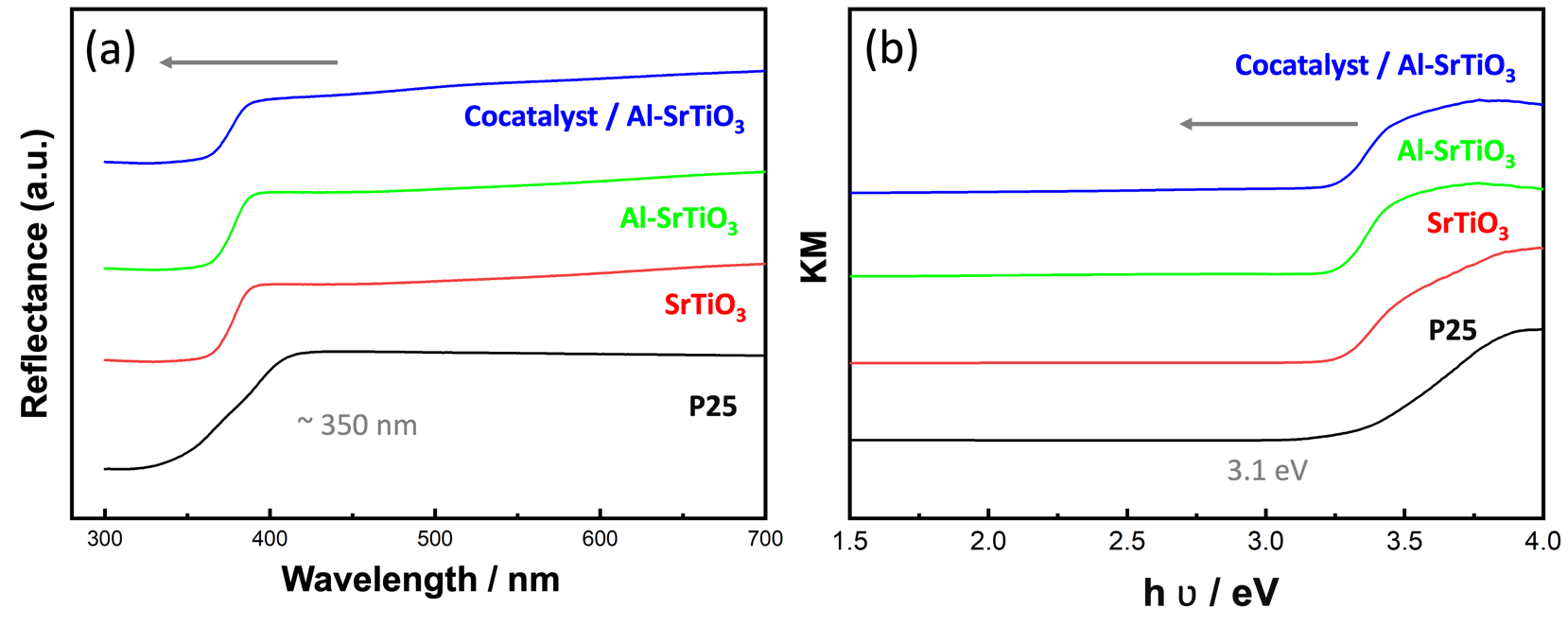


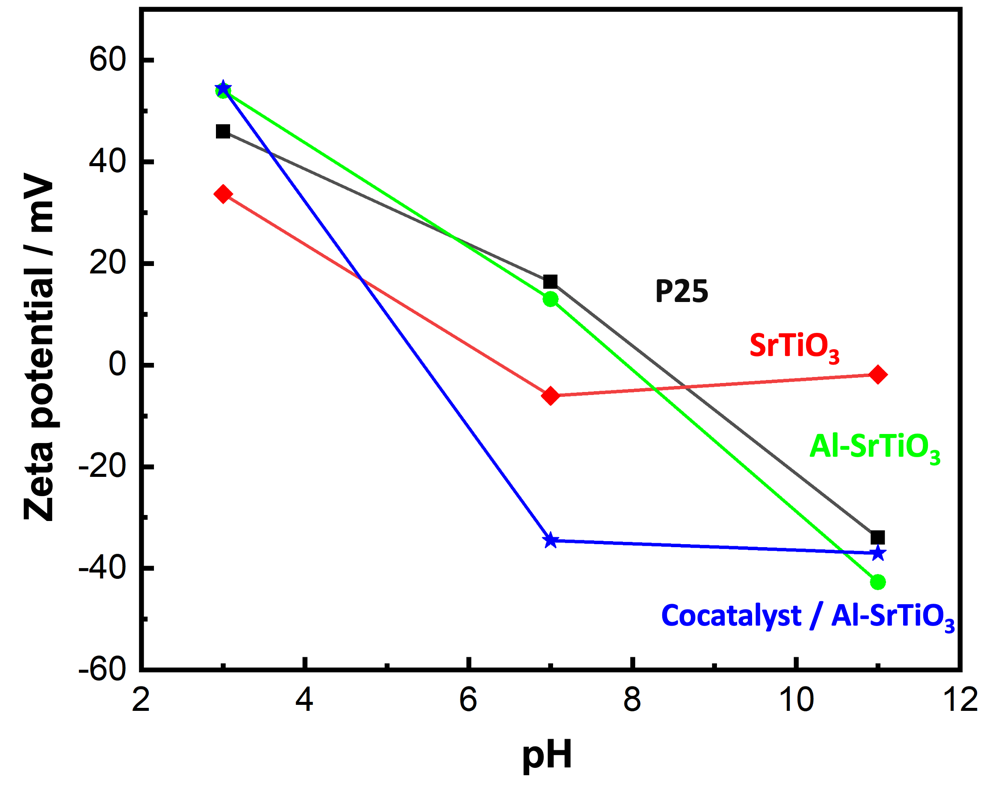
**S. Fig. 1:** a) UV-Vis. diffuse reflectance analysis of the investigated samples and b) the calculated bandgap values.

**S. Fig. 2:** Zeta potential at different pH values of the investigated samples.


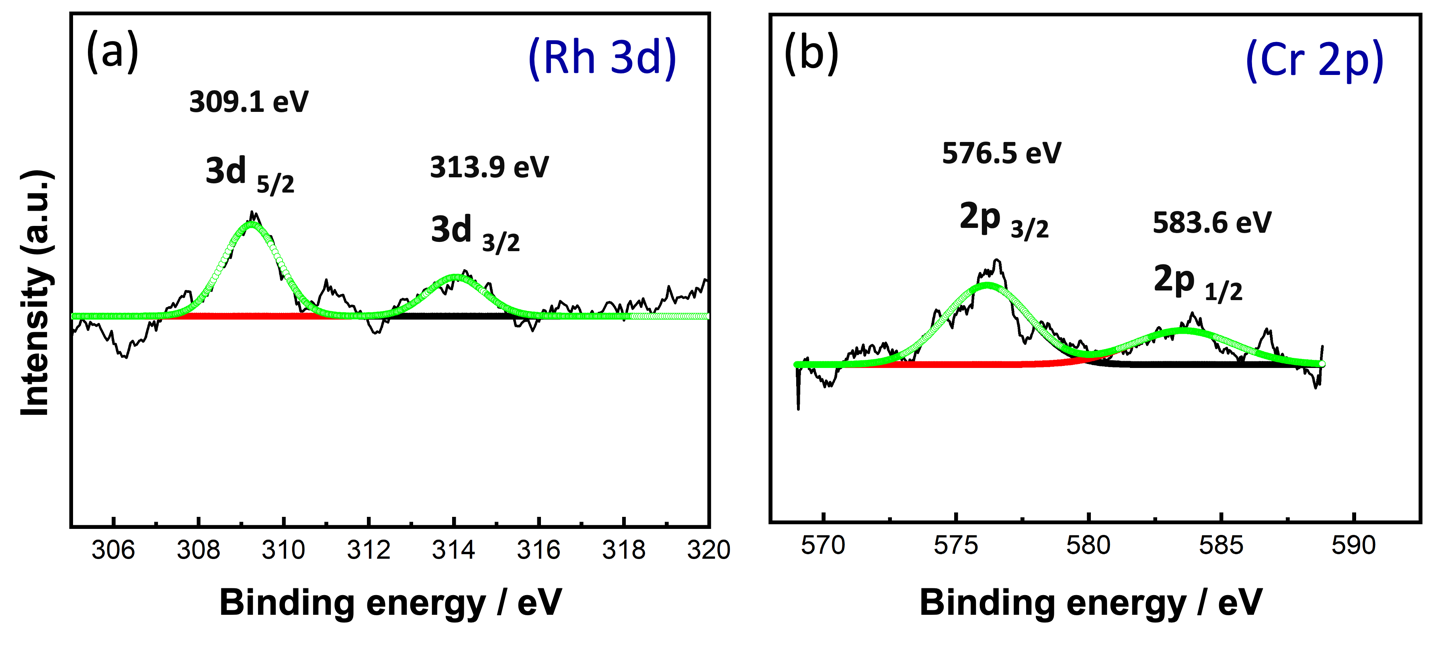


**S. Fig. 3:** deconvoluted XPS peak of a) Rh and b) Cr.


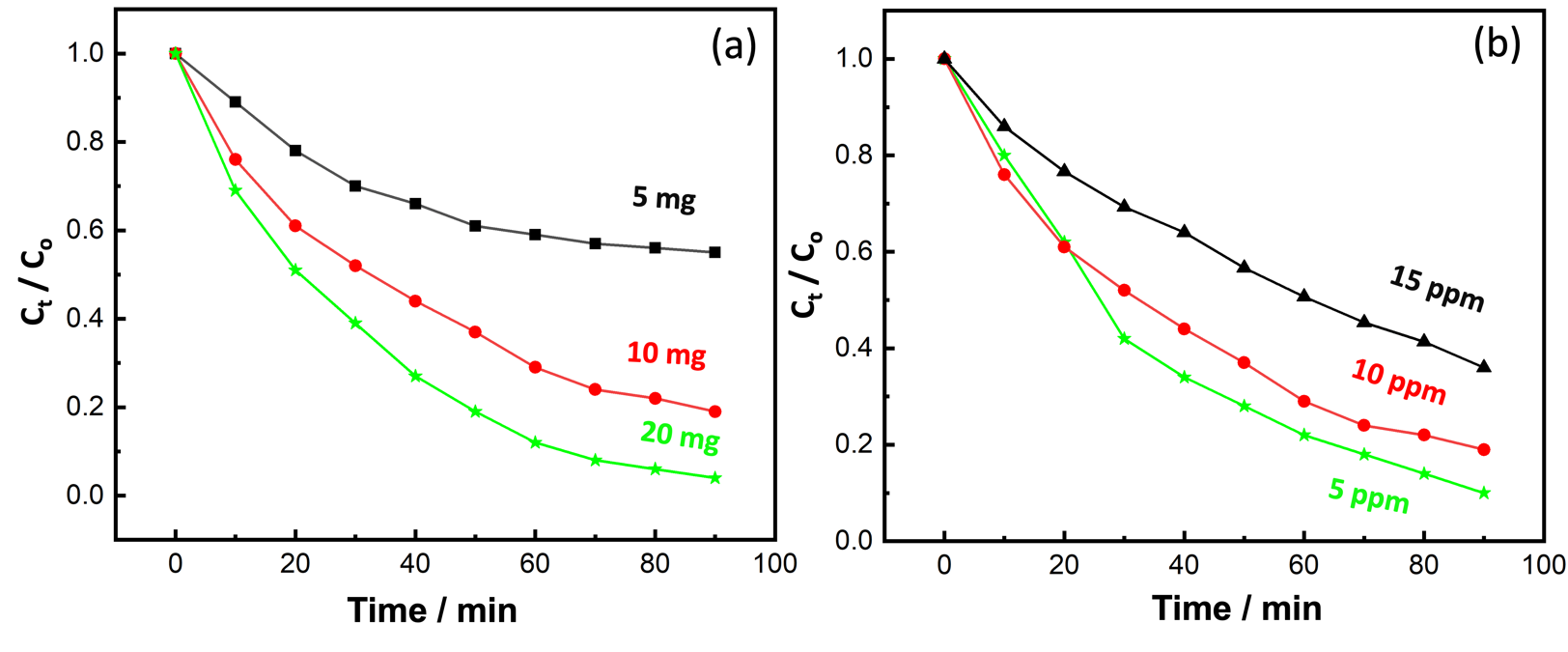


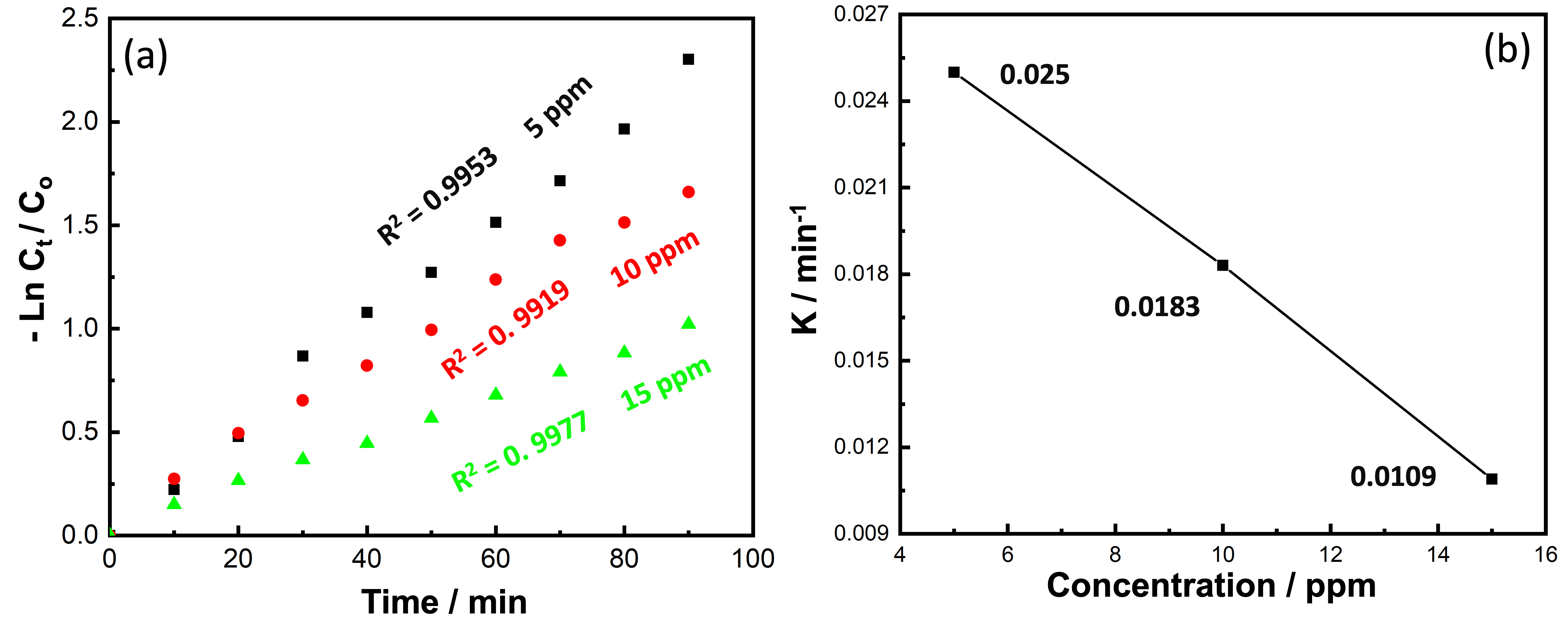
**S. F. 4:** (a) effect of photocatalyst dose and (b) effect of the initial concentration on CR degradation over cocatalyst loaded Al-SrTiO_3_ photocatalyst under UV radiation.

**S. Fig. 5:** (a) the relationship between -ln(C_t_/C_0_) and the time (t) for CR removal and (b) The rate constant k Vs. various initial concentrations of CR dye.


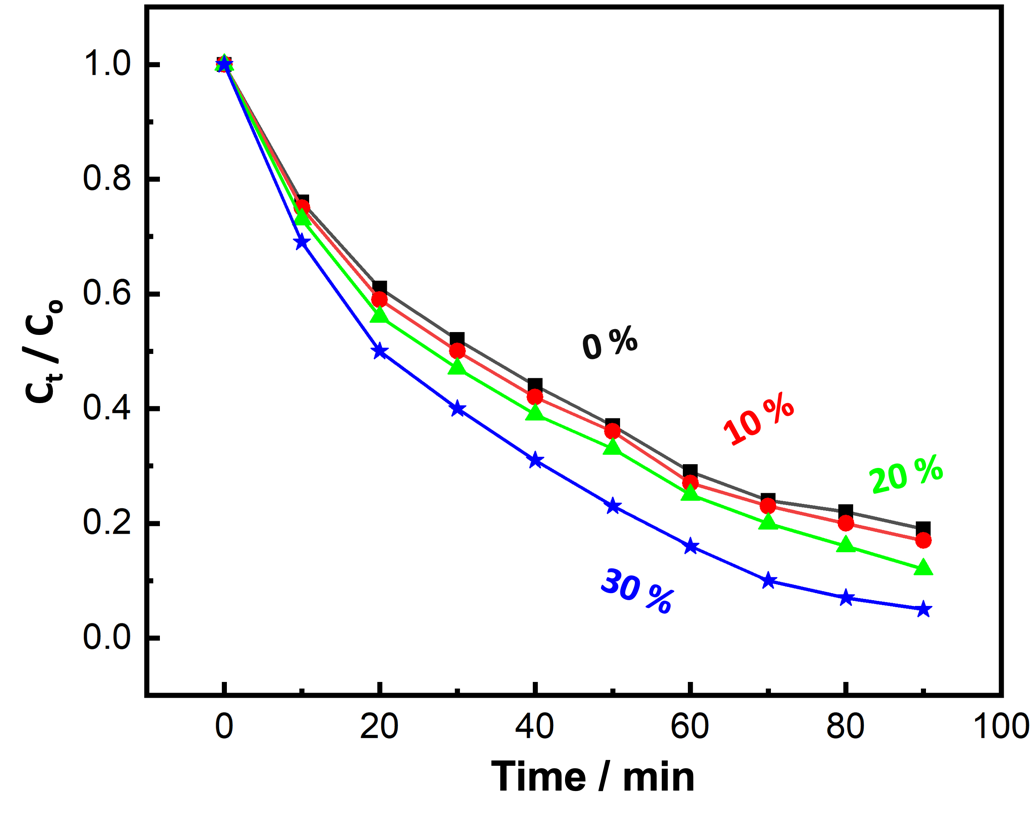


**S. Fig. 6:** effect of H_2_O_2_ on the removal percentage of CR over cocatalyst loaded Al-SrTiO_3_ photocatalyst.


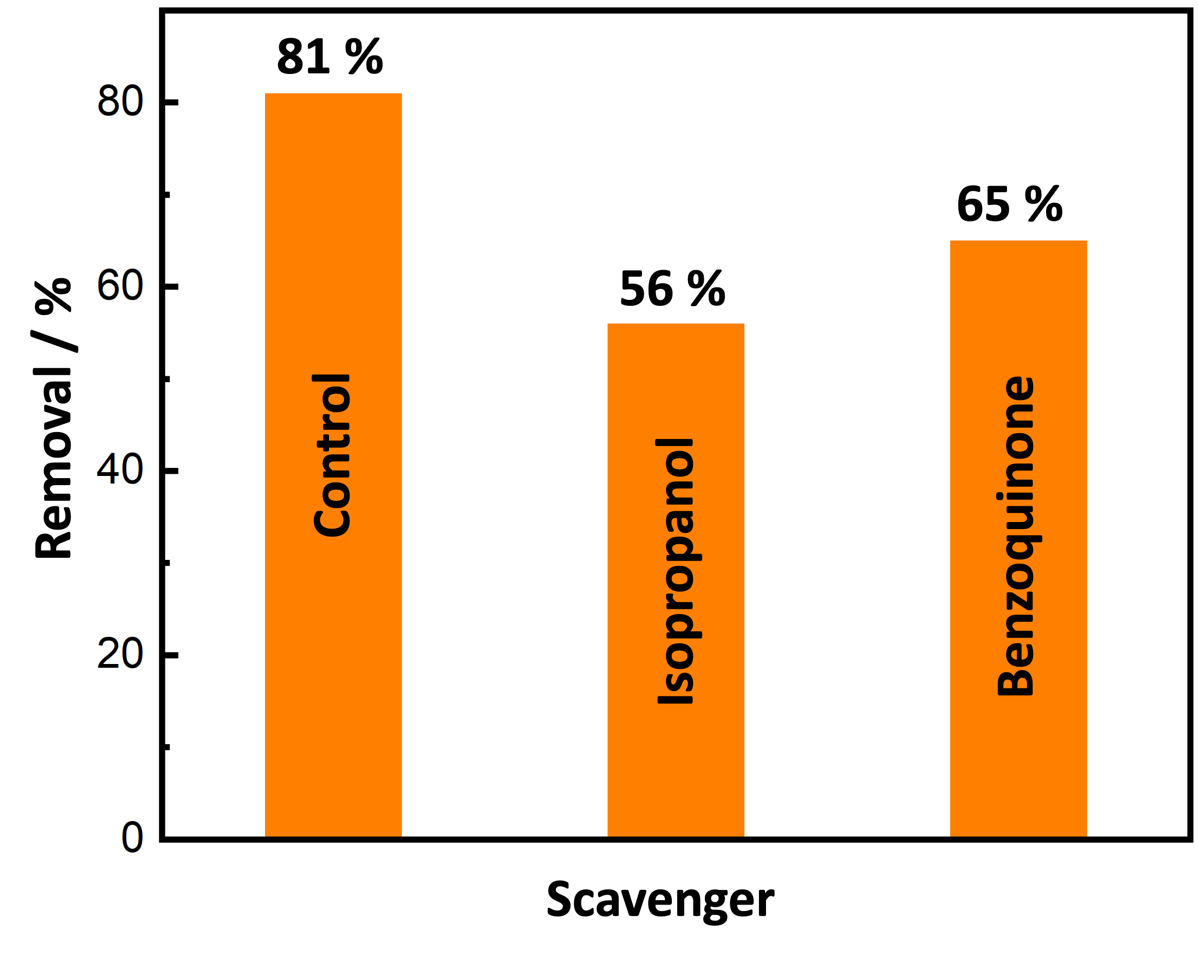


**S. Fig. 7:** Effect of scavengers on CR degradation over cocatalyst loaded Al-SrTiO_3_ sample.

**S. Fig. 8:** Proposed photocatalytic reaction mechanism of CR degradation by cocatalyst-loaded Al-SrTiO_3_ sample.
